# Determinants of neonatal mortality in the largest international border of Brazil: a case-control study

**DOI:** 10.1186/s12889-019-7638-8

**Published:** 2019-10-16

**Authors:** Suzana de Souza, Etienne Duim, Fernando Kenji Nampo

**Affiliations:** 1 Latin-American Institute of Life and Nature Sciences, Federal University of Latin-American Integration, 1000 Tarquínio Joslin dos Santos Ave ZIP, Foz do Iguassu, PR 85870-650 Brazil; 2 School of Public Health, Department of Epidemiology, University of Sao Paulo, 715 Dr. Arnaldo Ave ZIP, São Paulo, SP 01246-000 Brazil; 3 Evidence-Based Public Health Research Group, 1000 Tarquínio Joslin dos Santos Ave ZIP, Foz do Iguassu, PR 85870-650 Brazil

**Keywords:** Neonatal mortality, Determinants, Border region

## Abstract

**Background:**

Foz do Iguassu is a Brazilian municipality located in the most populous international border of the country and provides medical care to foreigners. Neonatal mortality in the city is higher than Brazil’s average and corresponds to 61% of all deaths in children under five. The current study aimed to identify the determinants of neonatal mortality in Foz do Iguassu.

**Methods:**

In this case-control study, we analyzed all neonatal deaths occurred in Foz do Iguassu from 2012 to 2016. Birth and mortality data were extracted from two national governmental databases (SINASC and SIM). We extracted data on (i) maternal sociodemographic characteristics, (ii) pregnancy care, and (iii) newborn characteristics. Multiple logistic regression with the conceptual framework was applied to examine the factors associated with neonatal mortality.

**Results:**

Most of the deaths occurred in the early neonatal period (65.9%). The factors associated with neonatal death were fetal congenital anomaly (OR 22.49; CI 95% 7.44–67.95; *p* = < 0.001); low birth weight (OR 17.15; CI 95% 8.56–34.37; *p* = < 0.001), first minute Apgar score under 7 (OR 15.60; CI 95% 8.23–29.67; *p* = < 0.001); zero to 3 prenatal appointments (OR 3.34; CI 95% 1.28–8.73; *p* = 0.014) and prematurity (OR 3.60; CI 95% 1.87–7.11; *p* = < 0.001).

**Conclusion:**

The high rate of neonatal death in Foz do Iguassu is strongly associated with newborn characteristics and not associated with maternal sociodemographic characteristics. Thus, the health services in the Brazilian side of this international borders should be aware of the quality of the prenatal care and childbirth attention provided.

## Background

The international border between Brazil, Paraguay, and Argentina is an area that includes Foz do Iguassu (Brazil), Ciudad del Este (Paraguay) and Puerto Iguazú (Argentina). Two international bridges connect these cities: the Tancredo Neves bridge, which connects Foz do Iguassu to Puerto Iguazu, and the Friendship International Bridge, which connects Foz do Iguassu to Ciudad del Este. Altogether, this is the most populous Brazilian border and covers a contingent of approximately 900,000 inhabitants, of which 264,044 live in Foz do Iguassu [1].

In Brazil, access to healthcare is granted by the Unified Health System (*Sistema Único de Saúde* - SUS), which provides care to all people in Brazil regardless of nationality and country of residence free of charge, differently than Argentina and Paraguay. Thus, the cross-border movement of patients between the three countries occurs mostly toward Brazil [2], which means that the health indicators found in Foz do Iguassu reflect in some way the healthcare available in the region.

The Infant Mortality Rate (IMR) quantifies the deaths of children under 1 year of age and is a classic indicator of the socioeconomic and health status of the population [3]. The IMR is divided into three periods: the early neonatal (death occurred within the first 7 days postpartum); late neonatal (death occurred from 8 to 27 days postpartum) and postneonatal (death occurred from 28 to 365 days postpartum). Brazil has made remarkable progress in facing child mortality, reducing IMR from 51/1000 in 1990 to 15/1000 live births in 2015 [4]. However, the current challenge is to minimize the Neonatal Mortality Rate (NMR) [5], which occurrence highlights the quality of prenatal care, childbirth, and newborn assistance [6] [7]. Although neonatal deaths worldwide account for 44% of the deaths in children under five [3, 8], in Foz do Iguassu, it represents 61% of those deaths [9].

The determinants of neonatal mortality are multiple and represent a complex interaction with sociodemographic, healthcare, and biological variables. For this reason, many authors have used a conceptual framework to explain how the interaction between several factors can result in death. Mosley and Chen [10] were pioneers in the application of this model to the study of child survival in developing countries. The framework is based on the premise that all sociodemographic and economic determinants of mortality necessarily operate through a standard set of biological mechanisms, or proximate determinants, to exert an impact of mortality. Thus, through a hierarchical structure, it is possible to consider and model distinct factors according to their precedence over time and their relevance to the outcome determination. The framework can be structured based on the context in which the outcome is studied, and variables can be allocated as many levels as needed to explain the outcome.

Despite the fact that the large proportion of neonatal deaths in Foz do Iguassu, its determinants remain unknown. Undoubtedly the lack of knowledge regarding the determinants of neonatal deaths precludes the diminishment of its occurrence or, at least, reduces the efficiency of actions promoted to decrease NMR. Furthermore, the better comprehension of the causes of newborn mortality at the most populated Brazilian international border may subsidize discussions about the challenges and possibilities of infantile healthcare in other international border areas within this country. Thus, this study aimed to identify the determinants of neonatal mortality in the Brazilian side of the Brazil-Paraguay-Argentina triple border from 2012 to 2016.

## Methods

### Study design

To identify the factors associated with neonatal death, we performed a case-control study.

### Setting

The study was conducted in Foz do Iguassu, with data from the period 2012 to 2016. The data were requested from the Municipal Health Department and delivered in Microsoft® Excel® spreadsheets.

### Participants

Cases were defined as all neonates who died within the first 27 days of life while controls were selected among those who survived the first 27 days of life. Neonates with a birth weight of fewer than 500 g or born before 24 gestational weeks were excluded. Four controls were matched to each case based on the date of birth.

### Data source

The Brazilian government implemented two health information systems to surveil the mortality and birth conditions, the Information System on Live Births (*Sistema de Informação Sobre Nascidos Vivos* - SINASC) in 1990 and the Information System on Mortality (*Sistema de Informação Sobre Mortalidade* - SIM) in 1975, respectively. All births and deaths occurring in Brazil, regardless of maternal nationality or living place, must be registered by filling out the “Declaration of Live Birth” and “Declaration of Death”, which populate the corresponding databases. Together, SINASC and SIM provide information that enables the construction of useful indicators for the management of health services in Brazil. The information gathered through these health information systems, which include maternal sociodemographic characteristics, information on pregnancy care and newborn characteristics, instigated the increase in research about maternal and child health in Brazil [11] [12].

Data used for the current study were extracted from SIM and SINASC databases. All neonatal deaths occurred in Foz do Iguassu from 2012 to 2016 were retrieved. The data were delivered in electronic spreadsheets individualized per year of occurrence and were later compiled into a single database for both SIM and SINASC.

To identify the births that evolved to neonatal death, we performed a probabilistic linkage to match SIM and SINASC registries. The use of linkage presupposes the existence of information recorded in standardized and individualized documents to allow the identification of the same individual in two or more databases. In this study, the linkage allowed us to merge the information contained in SIM and SINASC databases regarding maternal sociodemographic characteristics, information on pregnancy care, and newborn characteristics. The variables used to link the SIM and SINASC were the “Declaration of Live Birth” unique registry number, mother’s full name, birth date, and birth weight. The linkage was performed using Link Plus 2.0 software. Link Plus is a probabilistic record linkage program developed at CDC’s Division of Cancer Prevention and Control in support of CDC’s National Program of Cancer Registries (NPCR). Although designed to be used by cancer registries, this program can be used with any data in different contexts.

### Conceptual framework

**Fig. 1:**
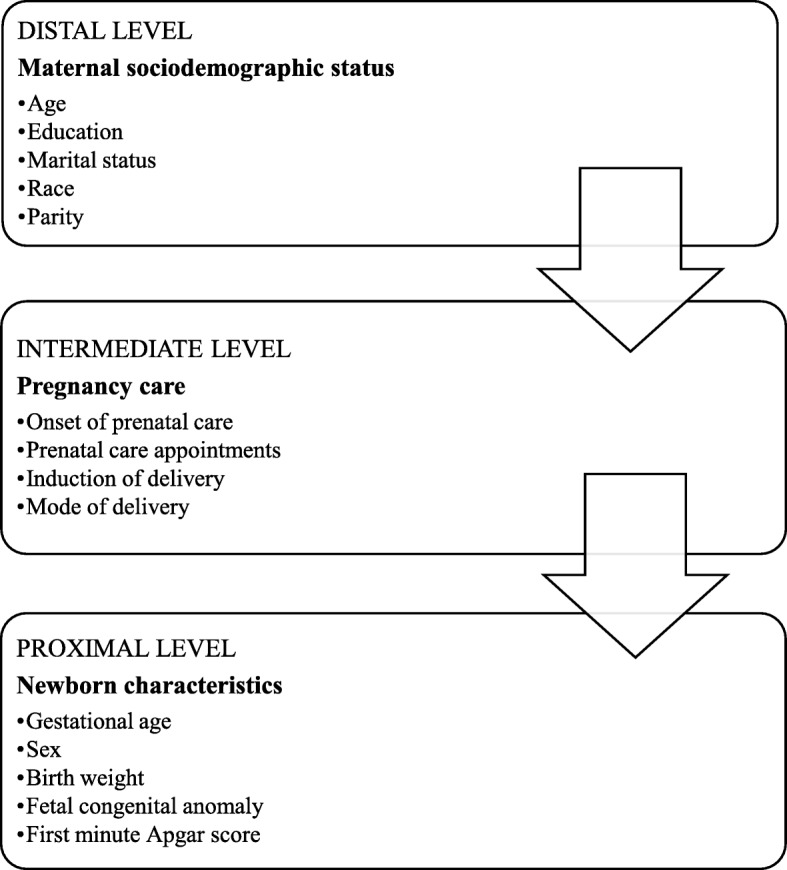
Conceptual framework for determinants of neonatal mortality in Foz do Iguassu

Based on other studies [13] [14], we modified the conceptual framework to our setting (Fig. 1) based on the available information in SIM and SINASC. We allocated the variables at three levels:

i. distal level: those variables inherent to the mother and more unlikely to be altered;
ii. intermediate level: those variables that may be affected by the distal level but at the same time may undergo interventions that may impact the outcome;
iii. proximal level: those variables related to the newborn that may Despite the fact that the outcome, and some of these variables may reflect the distal and intermediate levels.

### Variables

The information extracted from the databases and used in the conceptual framework included: Maternal age (less than 20 years (i.e. adolescent pregnancy), 20 to 34 years or more than 34 years (i.e. advanced maternal age)); mother’s education (up to 7 years of study, 8 to 11 years of study or > 12 years of study); marital status (with a partner or without a partner); race (white, yellow, black and brown); parity (primiparous or multiparous); onset of prenatal care (first trimester or after the first trimester); prenatal care appointments (0 to 3 appointments, 4 to 6 appointments or 7 or more appointments); induction of delivery (yes or not); mode of delivery (vaginal or cesarean section); gestational age, which is measured by the date of the last menstrual period, either by ultrasonography or both (preterm (up to 36 weeks), term (from 37 to 41 weeks) or post-term (42 weeks or more)); sex (male or female); birth weight (low weight (less than 2500 g;), adequate weight (2500–4000 g) or overweight (more than 4000 g)); fetal congenital anomaly (yes or no); first minute Apgar score (low (from 0 to 6 points) or high (from 7 to 10 points)).

As a remark, we merged the race ‘yellow’ with the race ‘white’ in the logistic model due to sample power.

### Statistical analyses

A multiple logistic regression model with hierarchical input of the variables in blocks was performed to verify the factors associated with neonatal mortality (Fig. 1). The Odds Ratio (OR) and 95% Confidence Intervals (95% CI) were calculated for each variable. In the final logistic regression model, the OR was adjusted for all the selected variables within that level and the variables of farther levels. In other words, at the distal level the OR of the maternal age, mother’s education, marital status, race and parity variables, were adjusted for each other; while at the intermediate level, the OR of the onset of prenatal care, prenatal care appointments, induction of delivery and mode of delivery variables, were adjusted for each other and for the independent variables of the distal level. Finally, at the proximal level, the OR of the gestational age, sex, birth weight, fetal congenital anomaly, and first minute Apgar score variables, were adjusted for each other and for the independent variables of both the intermediate and distal level.

The pairing process and statistical analyses were conducted using STATA 13.1 (Stata-Corp, College Station, TX, USA).

## Results

From 2012 to 2016, 25,563 births were registered in Foz do Iguassu, with an IMR of 15.45/1000 live births (*N* = 395). Overall, neonatal mortality counted for 71% (*N* = 280) of the infant deaths and represented an NMR of 10.95/1000 live births. Most of the neonatal deaths (65.9%; *N* = 147) occurred in the early neonatal period (i.e. within the first 7 days after delivery).

From the linkage between SIM and SINASC databases, 250 of the 280 “Declaration of Death” were successfully linked to their respective “Declaration of Live Birth”. After linkage, 19 birth records with gestational age less than 24 weeks and another 8 registries of birth weight lower than 500 g were excluded. The remaining 223 cases were paired with 889 controls that also met the inclusion criteria of gestational age and birth weight.

Among the distal level variables (i.e. maternal sociodemographics), cases, and controls presented quite similar characteristics on maternal age, education level, marital status, and race. In intermediate level (i.e. pregnancy care), the cases presented a higher frequency of prenatal care starting after the first trimester (22.4% versus 15.9%), pregnancies with zero to 3 prenatal care appointments (26.6% versus 6.8%), induction of delivery (79.7% versus 56.4%) and cesarean section (61% versus 56.7%). In the proximal level (i.e. newborn characteristics), the cases presented a higher occurrence of preterm newborn (49.7% versus 11%), low birth weight (72.2% versus 0.59%), fetal congenital anomaly (21.1% versus 1.2%) and low Apgar score (64.7% versus 5.2%) (Table 1).

**Table 1:** Characteristics of cases (*N* = 223) and controls (*N* = 889). Foz do Iguassu PR, 2012–2016

Variable	Cases N (%)	Controls N (%)
Distal Level		
Maternal age [mean (SD)]	26.6 (7.2)	26.7 (6.6)
Less than 19 years	44 (19.7)	130 (14.6)
From 20 to 34 years	144 (64.6)	639 (71.9)
Over 35 years	35 (15.7)	120 (13.5)
Education (in years of study)		
Less than 7 years	10 (4.5)	35 (3.9)
From 8 to 11 years	44 (19.7)	174 (19.6)
Over 12 years	169 (75.8)	679 (76.4)
Marital status		
With partner	101 (45.5)	448 (50.6)
Without partner	121 (54.5)	437 (49.4)
Race		
White	144 (64.6)	546 (61.7)
Black	6 (2.7)	19 (2.2)
Yellow	0 (0)	8 (0.9)
Brown	73 (32.7)	321 (35.2)
Parity		
Primiparous	95 (42.6)	346 (39)
Multiparous	128 (57.4)	540 (61)
Intermediate level		
Onset of prenatal care		
First trimester	152 (77.6)	742 (85.1)
After first trimester	44 (22.4)	129 (15.9)
Prenatal care appointments		
From 0 to 3 appointments	58 (26.6)	60 (6.8)
From 4 to 6 appointments	74 (33.9)	210 (23.7)
7 or more appointments	86 (39.5)	615 (69.5)
Induction of delivery		
No	44 (20.3)	384 (43.6)
Yes	173 (79.7)	497 (56.4)
Type of delivery		
Vaginal	87 (39)	385 (43.3)
Caesarean section	136 (61)	504 (56.7)
Proximal level		
Gestational age		
Preterm	148 (49.71)	94 (11)
Term	64 (30.2)	732 (85.6)
Post-term	0 (0)	29 (3.40)
Sex		
Female	103 (46.6)	424 (47.4)
Male	118 (53.4)	465 (52.3)
Birth weight		
Low weight	161 (72.2)	52 (0.59)
Adequate weight	59 (26.4)	783 (88.1)
Overweight	3 (0.1)	54 (0.6)
Fetal congenital anomaly		
Yes	47 (21.1)	11 (1.2)
No	176 (78.9)	878 (98.8)
First minute Apgar score		
High	77 (35.3)	841 (94.8)
Low	141 (64.7)	46 (5.2)

The multiple logistic regression using the conceptual framework revealed no maternal sociodemographic characteristics associated with neonatal death. Among the pregnancy care variables, induction of delivery that was associated with neonatal mortality in the logistic regression by blocks lost strength in the final model. Only the number of prenatal care appointments remained statistically associated with neonatal death (zero to 3 appointments; OR 3.34; CI 95% 1.28–8.73; *p* = 0.014). Most of the newborn’s characteristics were associated with neonatal death; except for the sex, all newborn’s characteristics presented a strong statistical association with neonatal mortality. Fetal congenital anomaly presented the strongest statistical association with neonatal death (OR 22.49; CI 95% 7.44–67.95; *p* = < 0.001), followed by low birth weight (OR 17.15; CI 95% 8.56–34.37; *p* = < 0.001), low first minute Apgar score (OR 15.60; CI 95% 8.23–29.67; *p* = < 0.001), and prematurity (OR 3.60; CI 95% 1.87–7.11; *p* = < 0.001) (Table 2).

**Table 2:** Factors associated with neonatal mortality based on conceptual framework analysis (distal, intermediate, and proximal factors). Foz do Iguassu PR, 2012–2016

Variable	OR	CI 95%	*p*-value	Adjusted OR	CI 95%	p-value
Distal Level						
Maternal age						
Less than 19 years	1.49	0.99–2.32	0.053	1.04	0.45–2.41	0.921
From 20 to 34 years	1.00	–	–	1.00	–	–
Over 35 years	1.23	0.79–1.93	0.349	0.95	0.43–2.11	0.905
Education (years of study)						
Less than 7 years	1.03	0.70–1.54	0.846	1.12	0.53–2.34	0.766
From 8 to 11 years	1.12	0.49–2.53	0.779	0.85	0.17–4.11	0.8
Over 12 years	1.00	–	–	1.00	–	–
Marital status						
With partner	1.00	–	–	1.00	–	–
Without partner	1.19	0.88–1.63	0.249	0.70	0.40–1.24	0.226
Race						
White	1.00	–	–	1.00	–	–
Black	1.16	0.48–3.10	0.519	0.91	0.16–5.20	0.912
Brown	0.90	0.66–1.23	0.681	0.90	0.50–1.64	0.742
Parity						
Primiparous	1.00	–	–	1.00	–	–
Multiparous	1.20	0.76–1.88	0.424	1.44	0.76–2.70	0.261
Intermediate level						
Onset of prenatal care						
First trimester	1.00	–	–	1.00	–	–
After first trimester	0.91	0.58–2.84	1.44	1.33	0.63–2.84	0.448
Prenatal care appointments						
0 to 3 appointment	6.76*	3.89–11.73	< 0.001	3.34*	1.28–8.73	0.014
4 to 6 appointments	2.63*	1.78–3.88	< 0.001	1.17	0.60–2.27	0.640
7 or more appointments	1.00	–	–	1.00	–	–
Induction of delivery						
No	3.84*	2.32–6.36	< 0.001	0.64	0.30–1.38	0.264
Yes	1.00	–	–	1.00	–	–
Type of delivery						
Vaginal	1.00	–	–	1.00	–	–
Caesarean section	0.77	0.49–1.23	0.289	1.83	0.88–3.80	0.106
Proximal level						
Gestational age						
Preterm	3.45*	1.86–6.39	< 0.001	3.64*	1.87–7.11	< 0.001
Term	1.00	–	–	1.00	–	–
Post-term	–	–	–	–	–	–
Sex						
Female	1.08	0.65–1.82	0.745	0.87	0.50–1.52	0.630
Male	1.00	–	–	1.00	–	–
Birth weight						
Low	18.34*	9.73–34.55	< 0.001	17.15*	8.55–34.37	< 0.001
Adequate	1.00	–	–	1.00	–	–
Overweight	0.93	0.21–4.06	0.928	0.98	0.22–4.44	0.994
Fetal congenital anomaly						
Yes	22.66*	8.78–58.47	< 0.001	22.49*	7.44–67.95	< 0.001
No	1.00	–	–	1.00	–	–
First minute Apgar score						
High	1.00	–	–	1.00	–	–
Low	14.51*	8.06–26.10	< 0.001	15.63*	8.23–29.67	< 0.001

* Variables statistically associated with neonatal death

## Discussion

According to our literature review, this study is the most comprehensive investigation regarding factors associated with neonatal mortality in an international border region. Previous studies in the Brazilian border region focused on the causes of infant mortality, including only Brazilian resident mothers [15] [16]. We opted to include all deaths, without distinction of maternal domicile, because the assistance to foreigners can affect the provision of services to the local population and also impacts the health indicators in the municipality. Additionally, the public health services in Foz do Iguassu impose several barriers to foreigners (e.g. preventing foreign patients from accessing the health services [2]), which is illegal and instigate the foreigners to falsely affirm that they live in Brazil; thus, the identification of the pregnant who live in Brazil would be imprecise. Moreover, our study focused on neonatal mortality because it is the main component of IMR and is the current challenge to reduce infant mortality [5].

In our investigation, the occurrence of congenital fetal anomaly presented the strongest statistical association with neonatal death, followed by low birth weight, low first minute Apgar score, zero to 3 prenatal appointments and preterm birth. As expected, our results indicated that the newborn’s characteristics are the most proximately related to neonatal mortality. Of note, all the statistically associated risk factors presented a moderate/strong association with neonatal death, with three of the five risk factors showing an OR above 15 [17].

There is consensus on the importance of prenatal care to minimize the risk of adverse outcomes for the mother and newborn [18] [19] [20]. The Parana’s Mother Network (*Rede Mãe Paranaense*) Program, a strategy implemented in the state of Parana, recommends seven prenatal appointments, preferably starting in the first trimester [19]. In the present study, only 39.5% of the cases performed the minimum number of recommended prenatal appointments, against 69.5% of the controls; however, caution is suggested in interpreting this result, because the low number of prenatal appointments may be associated with pregnancies of less than 30 weeks that would prevent the occurrence of 7 or more appointments. Births before the completion of 30 gestational weeks were more frequent among cases (42%) than among controls (0.3%).

The Apgar score shows the physiological conditions and responsiveness of the newborn to extrauterine life and has been used for more than 50 years as an useful and low-cost tool that remains relevant to contemporary practice in predicting the risk of newborn death and, consequently, to identify those who need additional care in the neonatal and post-neonatal period [21] [22]. The first minute Apgar score below 7 was statistically associated with neonatal mortality in our study and half of the deaths also presented low Apgar score, corroborating other studies [23] [24] [25]. Despite being considered an endpoint, the low Apgar score precedes death and can be prevented, especially with adequate attention to pregnant women. We believe that Intrapartum asphyxia demands specific attention during delivery and the prevention of problems related to intrauterine hypoxia and timely interventions may decrease the risk of death due to intrapartum asphyxia in Brazil, considering that almost 100% of births occur in health institutions [5] [26].

Prematurity is one of the leading causes of child death in the world [27] [28]. The gestational age, which was statistically associated with neonatal mortality in this study, is a relevant factor because it is associated with low birth weight, incomplete fetal development, and low Apgar score. The supply of oxygen through the placenta increases with the course of gestation according to the fetal needs [21] [24]; a premature birth interrupts this process, exposing the newborn to oxygen deprivation, which can be harmful to the tissues, especially the nervous system [29], increasing the risk of cerebral palsy, visual disturbances and chronic disease in adulthood [30]. Consequently, a premature newborn increases intensive care expenses, and also affects the family’s social, financial, and emotional status. Therefore, investing in prenatal care, focusing on the identification of pregnancies at risk of prematurity may reduce both the financial burden and the social impact of this event.

For this study, all congenital anomalies recorded in SINASC were included according to their code of Chapter 17 of the 10th International Classification of Diseases. The strong association between congenital fetal anomaly and neonatal death observed in this study corroborates the evidence found in other studies [12] [19]. Congenital anomalies contribute significantly to premature birth, morbidity, and neonatal mortality. Since the improvement of healthful conditions and the reduction of infant deaths due to infectious and parasitic diseases, the anomalies became an important risk factor associated to IMR, evidencing the process of epidemiological transition that most countries experience. According to an a posteriori analysis, most newborns with congenital anomalies were born at normal gestational age (76%) and normal birth weight (78%). Therefore, the only risk factor considered to be unavoidable seems not to be related to prematurity and low birth weight, indicating that it may be possible to reduce neonatal mortality by acting on avoidable risk factors [31].

Maternal sociodemographic factors can impact childcare, not only about access to health services but also living conditions, basic sanitation, and nutrition [32]. Although previous studies have shown negative effects of maternal age and education on perinatal outcomes [33] [34], in our study, those variables were not associated with neonatal mortality. The lack of association between maternal sociodemographic characteristics and neonatal death may be related to the fact that two-thirds of the deaths occurred in the early neonatal period when most mothers and newborns are being assisted in hospitals, suggesting poor healthcare provided in the early postpartum period, regardless of sociodemographic characteristics. At least partially, this poor healthcare provided in the early neonatal period may be related to hospital overload. In Foz do Iguassu, the only hospital with a Neonatal Intensive Care Unit (NICU) is a reference for maternity to 9 municipalities within western Parana; additionally, it receives pregnant women and newborns from neighboring countries. Nevertheless, at the end of this research, the hospital had only 18 intensive or intermediate care unit beds, which is 25% below the established by the Brazilian Ministry of Health.

In an a posteriori investigation, we found that the rates of prematurity, low birth-weight, low first minute Apgar score, and congenital anomaly incidence are not worse than those found in Parana state and Brazil. In contrast, Foz do Iguassu presents twice the rate of unsatisfactory prenatal appointments in comparison to Parana state and Brazil. Thus, the high rates of neonatal deaths in this region possibly translate the poor quality of the local health services.

Although some variables of the proximal level, such as Apgar score, birth-weight, and prematurity, are considered endpoints, we understand that they precede death and reflect distal and intermediate level variables, being relevant in the model constructed for this study. Besides, they are risk factors for neonatal death already established in the literature. With this in mind, we proposed the analysis of these risk factors through the conceptual framework, which analyzes the effect of each variable on neonatal death hierarchically mediated by the other variables.

We acknowledge some limitations in this research. Firstly, as every study that relies on secondary data analysis, missing or incorrect information is an inherent risk. Also, the official databases used in our research do not provide information on maternal diseases, which may also be associated with neonatal mortality. Regarding the strengths of the study, to improve the reliability of our data, we performed an a priori data completeness analysis for the period from 1996 to 2016. Considering the high frequency of missing data before 2012 and the modifications in the data gathered through SINASC in 2011, we analyzed only births occurred from 2012 to 2016. Besides, the choice of analysis through the conceptual framework allowed us to model different factors according to their precedence over time and their relevance to the outcome determination; moreover, this analysis model represents a strategy for dealing with a large number of conceptually related variables present in epidemiological studies.

## Conclusion

Fetal congenital anomaly, low birth-weight, low first minute Apgar score, zero to 3 prenatal appointments, and prematurity are associated with neonatal death. At the largest international border of Brazil, the maternal sociodemographic condition is not associated with neonatal mortality. The implications for practice that may be derived from our study is that health services need to (i) increase the prenatal surveillance and (ii) pay attention to its in-patient care for pregnant women and newborns. Finally, based on our findings, we recommend that future research investigates (i) the quality of prenatal care, childbirth and early postpartum attention, (ii) the risk factors associated with prematurity, (iii) the impact of cross-border patients on childbirth indicators and (iv) the epidemiology of congenital anomaly in this international border.

## Data Availability

The datasets analyzed during the current study are not publicly available due to the privacy policy imposed by the Brazilian government but may be available from the corresponding author on reasonable request.

## Abbreviations

IMR: Infant Mortality Rate
NICU: Neonatal Intensive Care Unit
NMR: Neonatal Mortality Rate
SIM: Information System on Mortality (Sistema de Informação de Mortalidade)
SINASC: Information System on Live Births (Sistema de Informação de Nascidos Vivos)
